# Melanin production in coelomycetous agents of black grain eumycetoma

**DOI:** 10.1093/trstmh/traa168

**Published:** 2021-01-19

**Authors:** Wilson Lim, Florianne Parel, Sybren de Hoog, Annelies Verbon, Wendy W J van de Sande

**Affiliations:** Erasmus MC , University Medical Center Rotterdam, Department of Microbiology and Infectious Diseases, Rotterdam, the Netherlands; Erasmus MC , University Medical Center Rotterdam, Department of Microbiology and Infectious Diseases, Rotterdam, the Netherlands; Center of Expertise in Mycology of Radboud University Medical Center/Canisius Wilhelmina Hospital, Nijmegen, the Netherlands; Erasmus MC , University Medical Center Rotterdam, Department of Microbiology and Infectious Diseases, Rotterdam, the Netherlands; Erasmus MC , University Medical Center Rotterdam, Department of Microbiology and Infectious Diseases, Rotterdam, the Netherlands

**Keywords:** *Falciformispora senegalensis*, *Falciformispora tompkinsii*, *Medicopsis romeroi*, melanin, mycetoma, *Trematosphaeria grisea*

## Abstract

**Background:**

Eumycetoma is a fungal infection characterised by the formation of black grains by causative agents. The melanin biosynthetic pathways used by the most common causative agents of black-grain mycetoma are unknown and unravelling them could identify potential new therapeutic targets.

**Method:**

Melanin biosynthetic pathways in the causative fungi were identified by the use of specific melanin inhibitors.

**Results:**

In *Trematosphaeria grisea* and *Falciformispora tompkinsii*, 1,8-dihydroxynaphthalene (DHN)-melanin synthesis was inhibited, while DHN-, 3,4-dihydroxyphenylalanine (DOPA)- and pyo-melanin were inhibited in *Medicopsis romeroi* and *Falciformispora senegalensis.*

**Conclusion:**

Our data suggest that *Me. romeroi* and *F. senegalensis* synthesise DHN-, DOPA- and pyo-melanin, while *T. grisea* and *F. tompkinsii* only synthesise DHN-melanin.

## Introduction

Eumycetoma is a subcutaneous fungal disease characterised by the presence of black, white or occasionally yellow grains in infected tissue.^1^ It is recognised as a neglected tropical disease by the WHO and can be caused by more than 40 different fungal species.^1^ Four of the five most prevalent eumycetoma-causing agents form black grains, namely, *Madurella mycetomatis, Falciformispora senegalensis, Trematosphaeria grisea* and *Medicopsis romeroi*, while the fifth, *Scedosporium boydii*, forms white grains.^1^ Melanin was found to be responsible for the black colour in *Ma. mycetomatis*.^2^ Melanins are hydrophobic, negatively charged macromolecular pigments formed by oxidative polymerisation of phenolic or indolic compounds. They contribute to virulence and play a role in the protection against various environmental stresses, antifungal agents and host defences. It is known that melanised fungi are often more difficult to treat and have more relapses compared with non-melanised fungi.^3^

**Figure 1.**
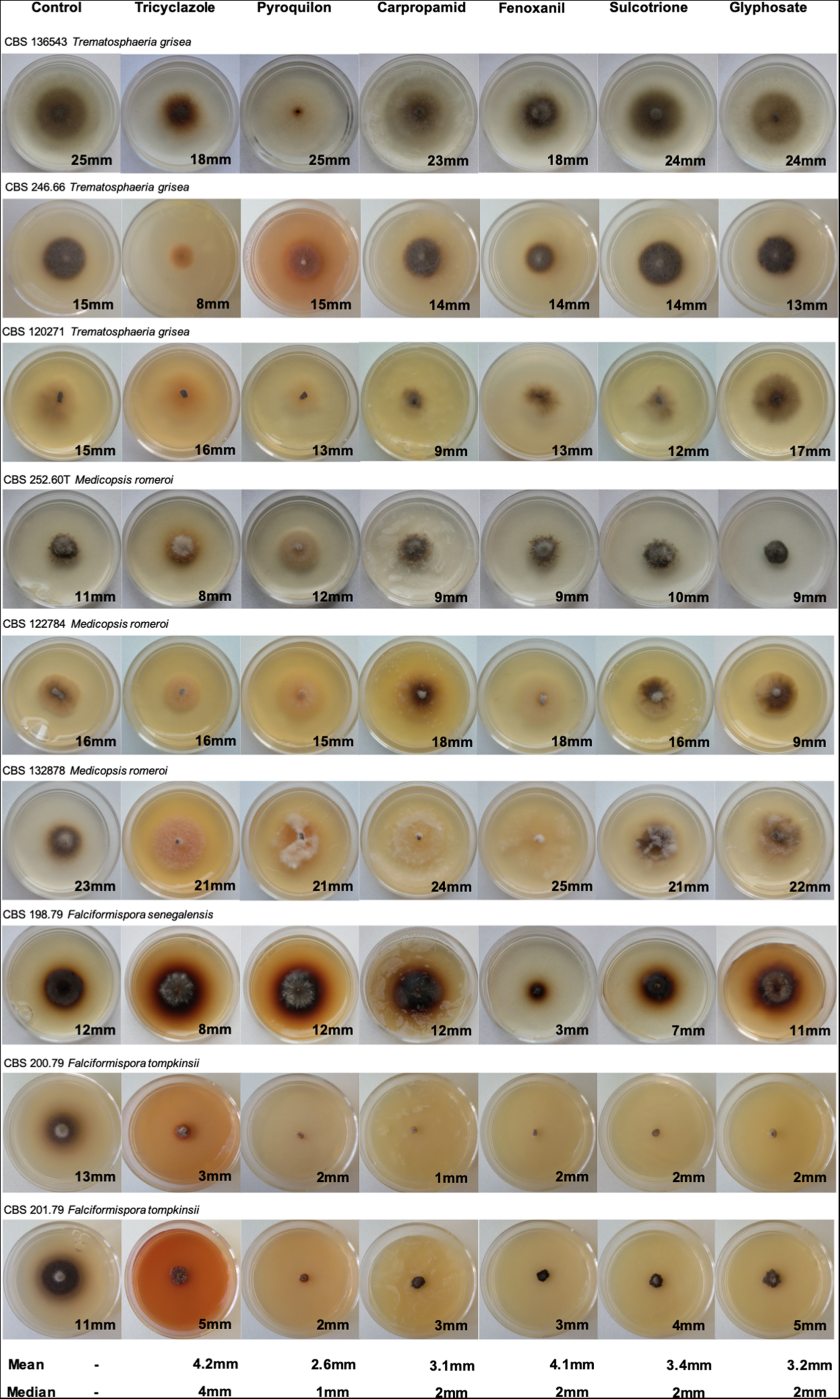
The effect of DHN-, DOPA- and pyo-melanin inhibitors in melanin inhibition and fungal growth in *T. grisea, Me. romeroi, F. senegalensis* and *F. tompkinsii.* DHN-melanin inhibitors, tricyclazole, pyroquilon, carpropamid and fenoxanil; DOPA-melanin inhibitor, glyphosate; pyo-melanin inhibitor, sulcotrione. The melanin inhibitors used in each column are depicted at the top of the figure. The mean and median in growth inhibition by each inhibitor is depicted at the bottom of the figure in the same column. Growth expansion (by diameter) is depicted at the bottom right of each fungus inhibited by the respective melanin inhibitor. Melanin inhibition was defined when a reduction in black pigmentation or production of orange pigments was observed in fungi treated with the inhibitors. Tricyclazole and pyroquilon inhibited melanin synthesis and reduced fungal growth in all isolates. Glyphosate and sulcotrione inhibited melanisation in *Me. romeroi* and *F. senegalensis*. All inhibitors were tested on the isolates at 50 mg/L except for tricyclazole and pyroquilon in CBS198.79 and fenoxanil in CBS200.79. Those were tested at 5 mg/L as 50 mg/L completely inhibited fungal growth.

Three melanin biosynthesis pathways have been described in fungi: the 1,8-dihydroxynaphthalene (DHN)-, the 3,4-dihydroxyphenylalanine (DOPA)- and the pyo-melanin biosynthetic pathways. Interference of DHN-melanin synthesis can be achieved either by inhibiting tetrahydroxynaphthale reductase with tricyclazole (TCZ) and pyroquilon (PYR), or by inhibiting scytalone dehydratase with carpropamid (CAR) and fenoxanil.^2,4^ Glyphosate (GLY) inhibits DOPA-melanin, while sulcotrione (SCT) inhibits pyo-melanin.^2^ We have shown that *Ma. mycetomatis* forms melanin via DHN- and pyo-melanin pathways^2^; however, the pathways used in *F. senegalensis, T. grisea* and *Me. romeroi* are still unknown. To understand the types of melanin involved in grain synthesis, we used melanin inhibitors to determine the melanin biosynthetic pathways present in these prevalent agents of human eumycetoma, and in the related, less common agent of eumycetoma, *F. tompkinsii.*

## Materials and methods

All the melanin inhibitors were obtained from Sigma-Aldrich (Sigma-Aldrich Chemie NV, Zwijndrecht, the Netherlands) and were dissolved in Dimethyl sulfoxide (DMSO). Three *T. grisea*, three *Me. romeroi*, two *F. tompkinsii* and one *F. senegalensis* were cultured on minimal media supplemented with 0.5, 5 or 50 mg/L melanin inhibitors as described

earlier.^2^ Fungi were cultured for 7 wk at room temperature or at 37°C. Melanin inhibition was determined visually on the basis of morphological and colour differences compared with the control on the same medium without inhibitors. Melanin inhibition was defined as a reduction of black pigmentation, or as production of orange pigments on media with inhibitor. Fungal growth rates were determined by the diameter of the colony.

## Results and discussion

To determine the types of melanin involved in grain synthesis, melanin inhibition was performed on *F. senegalensis, T. grisea, Me. romeroi* and *F. tompkinsii.* Our data revealed that all four species were inhibited by DHN-melanin inhibitors, similar to *Ma. mycetomatis* in earlier studies (Figure 1).^2^*Trematosphaeria grisea* and *F. tompkinsii* were inhibited by TCZ and PYR, while *Me. romeroi* and *F. senegalensis* were inhibited in two steps of the DHN-melanin pathway, as observed in TCZ, PYR, CAR and PYR inhibition. An orange hue was observed in isolates inhibited by TCZ and PYR due to the production of coloured by-products in the melanin biosynthesis pathway such as flaviolin and juglone.^4^ Inhibition in expansion growth was also noted. The highest reduction in growth was observed when DHN-melanin was inhibited by TCZ (median 4 mm) (Figure 1).

In addition to DHN melanin, *Ma. mycetomatis* also forms pyo-melanin. Using the pyo-melanin inhibitor SCT, a slight reduction of colony darkness and diameter was noted in *Me. romeroi* and *F. senegalensis*, while no reduction in melanisation or expansion growth was observed in *T. grisea* and *F. tompkinsii*, indicating that only *Me. romeroi* and *F. senegalensis* formed significant amounts of pyo-melanin. *Medicopsis romeroi* and *F. senegalensis* were also the only species inhibited by the DOPA-melanin inhibitor GLY. A diffusible pigment was also present in *Me. romeroi* inhibited with GLY, similar to what was observed in isolates inhibited by TCZ and PYR (Figure 1). Consequently, the pathways of melanin production were concluded to be species-dependent; in summary, DHN-melanin in *T. grisea* and *F. tompkinsii* and DHN-, DOPA- and pyo-melanin in *Me. romeroi* and *F. senegalensis.* Remarkably, sibling species *F. tompkinsii* and *F. senegalensis* differ in the presence of melanin pathways even although they are phylogenetically close, as shown by similarity in their rDNA Internal transcribed spacer (ITS) gene.^5^ The ITS sequences of *F. senegalensis* CBS198.79 are 86.03% (490 out of 580 bp) and 86.06% (500 out of 580 bp), identical to those of *F. tompkinsii* isolates CBS200.79 and CBS201.79, respectively. In *F. senegalensis*, pyo-melanin and DOPA-melanin were present, while in *F. tompkinsii* these were absent or present below the detection level of the applied method.

Since the analysed fungi are able to synthesise melanin using multiple pathways, inhibition of a single pathway is not sufficient to completely stop melanin production. To determine if complete eradication of melanin pigmentation of the mycelium can be achieved by simultaneous inhibition of multiple biosynthetic pathways, the strains were subjected to four combined inhibition protocols, namely, inhibition of DHN and DOPA (PYR and GLY), pyo-melanin and DOPA (SCT and GLY), DHN and pyo-melanin (PYR and SCT), and DHN-, pyo- and DOPA-melanin (PYR, SCT and GLY). No enhanced melanin inhibition was noted compared with inhibition by single inhibitors. It may be hypothesised that these inhibitors alone were not enough to completely inhibit their respective melanin-biosynthesis pathways, leading to melanin production via a different route.

A clear limitation of this study is that our data were derived from only nine fungal isolates. While these nine fungal isolates do not reflect all eumycetoma-causing agents, there is unfortunately no larger collection of these fungi currently available in the world. With a larger collection also consisting of other eumycetoma-causing agents, we may be able to obtain a clearer understanding of the types of melanin involved in grain synthesis.

DHN-melanin is known to be present in *Ma. mycetomatis*^2^ and was found in all the mycetoma agents tested here. Since a reduction in expansion growth was noted with some of the DHN-melanin inhibitors and DHN-melanin is the prevalent type of melanin in all fungi investigated, it could be explored as a potential drug target for some of the causative agents. An interesting approach might be to enhance the efficacy of azole therapy for mycetoma since the presence of melanin is known to lead to decreased drug susceptibility. In *Ma. mycetomatis*, melanin was able to bind to itraconazole, the drug currently in use in mycetoma treatment and lower inhibitory concentrations.^2^ A similar phenomenon has also been observed with *Aspergillus fumigatus* DHN-melanin.^2^ Since DHN-melanin is present in all the causative agents tested here, it could be envisioned that similar protection would occur in these species as well. Therefore, to enhance the efficacy of the azoles currently used in mycetoma treatment, combination treatment with melanin inhibitors should be investigated.

## Data Availability

Data available upon request from corresponding author.

## References

[1] van de Sande WW . Global burden of human mycetoma: a systematic review and meta-analysis. PLoS Negl Trop Dis. 2013;7(11):e2550. 24244780 10.1371/journal.pntd.0002550 PMC3820768

[2] van de Sande WW , de Kat J, Coppens J et al. Melanin biosynthesis in Madurella mycetomatis and its effect on susceptibility to itraconazole and ketoconazole. Microbes Infect. 2007;9(9):1114–23. 17644456 10.1016/j.micinf.2007.05.015

[3] Brandt ME , Warnock DW. Epidemiology, clinical manifestations, and therapy of infections caused by dematiaceous fungi. J Chemother. 2003;15(Suppl 2):36–47. 14708965 10.1179/joc.2003.15.Supplement-2.36

[4] Cunha MM , Franzen AJ, Alviano DS et al. Inhibition of melanin synthesis pathway by tricyclazole increases susceptibility of Fonsecaea pedrosoi against mouse macrophages. Microsc Res Tech. 2005;68(6):377–84. 16358282 10.1002/jemt.20260

[5] Ahmed SA , van de Sande WW, Stevens DA et al. Revision of agents of black-grain eumycetoma in the order Pleosporales. Persoonia. 2014;33:141–54. 25737597 10.3767/003158514X684744 PMC4312930

